# Optical Coherence Tomography Angiography Parameters in Indian Patients With Central Serous Chorioretinopathy

**DOI:** 10.7759/cureus.46467

**Published:** 2023-10-04

**Authors:** Punita K Sodhi, Kavya C Rao, Archana T R, Akanksha Gautam, Divya D, Aman S Rana, Rajesh Kumar, Sahadev Santra, Avilasha Mohapatra

**Affiliations:** 1 Ophthalmology, Guru Nanak Eye Centre, Maulana Azad Medical College, New Delhi, IND; 2 Community Medicine, Maulana Azad Medical College, New Delhi, IND

**Keywords:** chorio-capillaries, vascular density, superficial capillary plexus, optical coherence tomography angiography, optical coherence tomography, foveal avascular zone, deep capillary plexus, choroid, central serous chorioretinopathy

## Abstract

Background

In this study, we aimed to evaluate optical coherence tomography angiography (OCTA) parameters among Indian patients affected with central serous chorioretinopathy (CSCR).

Methodology

A cross-sectional study on Indian patients having unilateral or bilateral affection with CSCR was conducted at the Department of Ophthalmology, Guru Nanak Eye Centre, and Maulana Azad Medical College, New Delhi. A history of ocular symptoms such as a diminution of vision, metamorphopsia, decreased contrast sensitivity (CS), and defective color vision (CV) and their duration were obtained. A detailed ocular examination for best-corrected visual acuity (BCVA), intraocular pressure (IOP), CV, and CS was done. Following this, fundus fluorescein angiography (FFA) and indocyanine green angiography (ICGA) were performed. OCT was done for central foveal thickness (CFT), subfoveal choroidal thickness (SFCT), neurosensory detachment (NSD), pigment epithelial detachment (PED), and choroidal neovascular membranes (CNVMs). The OCTA imaging was done to examine the foveal avascular zone (FAZ) size, perimeter and circularity, vessel density (VD), and features such as enlarged/distorted FAZ, dark areas, dark spots, abnormal vessels, and choriocapillaris island (CCI) in the retino-choroidal layers. We compared the OCTA features of affected eyes with those of fellow eyes.

Results

The study involved 52 eyes of 40 CSCR patients, including 32 (80%) males and eight (20%) females with a mean age of 39.3 ± 6.1 (24-49) years. Of the 40 patients, 12 (30%) had a bilateral involvement. The mean CFT was 300.3 ± 158.4 µ, and the SFCT was 258.5 ± 60.4 µ. The mean distance BCVA was the logarithm of the minimum angle of resolution (logMAR) 0.58 ± 0.32. The OCTA showed features such as enlarged/distorted FAZ (36.53% eyes), dark areas (NSD/PED) (84.61% eyes), dark spots (PED) (5.76% eyes), abnormal vessels (dilated vessels/CNVM) (96.15% eyes), and CCI (17.30% eyes). The mean FAZ area, perimeter, and circularity were 0.40 ± 0.71 mm^2^, 41.8 ± 280.0 mm, and 0.48 ± 0.12, respectively. The VD in the superficial capillary plexus (SCP) was 25.4 ± 14.1, deep capillary plexus (DCP) 15.0 ± 11.5, outer retina (OR) 5.9 ± 6.8, outer retinal choriocapillaris (ORCC) 33.7 ± 16.9, choriocapillaris 29.7 ± 17.5, and choroid 29.9 ± 17.5. The fellow eyes showed a mean FAZ area, perimeter, and circularity of 0.34 ± 0.23 mm^2^, 76.8 ± 391.2 mm, and 0.47 ± 0.11, respectively, while VD of SCP was 25.9 ± 13.6, DCP 16.5 ± 11.7, OR 14.3 ± 14.9, ORCC 38.0 ± 16.5, choriocapillaris 36.3 ± 17.7, and choroid 35.5 ± 19.2.

Conclusions

The CSCR eyes had a thicker fovea and sub-foveal choroid (SFC). The FAZ area of affected eyes was larger, while the perimeter was smaller than that in the fellow eye. In the affected eye, the VD in all the retino-choroidal layers was lower, although it was significantly reduced in OR whole (p = 0.006) and foveal choroid (p = 0.022).

## Introduction

Central serous chorioretinopathy (CSCR) is characterized by serous retinal detachment (RD) in the macular area. The diagnosis of CSCR is established through a presentation that includes a painless decrease in central vision, central or paracentral scotoma, metamorphopsia, micropsia, and disturbed color vision. It predominantly affects males, with an age of onset between 30 and 50 years, associated with a type A personality or systemic steroid therapy [1].

Leaking points are observed on fundus fluorescein angiography (FFA). The indocyanine green angiography (ICGA) shows hyperflourescence corresponding to these leaking points and the areas of choriocapillaris hyperpermeability [2]. In the affected eyes, optical coherence tomography (OCT) shows a serous detachment of the retina in the foveal region, often accompanied by retinal pigment epithelium (RPE) detachment and a thickened sub-foveal choroid (SFC). OCT angiography (OCTA) is a new technique to examine the macula and depict retinal and choroidal vessels qualitatively and quantitatively in an en-face high-resolution vascular imaging. The technique delineates the foveal avascular zone (FAZ) and shows vessel density (VD) as an area occupied by vessels (white pixels) divided by the total image area (white and black pixels). OCTA can detect vascular abnormalities of CSCR as high-intensity signals and shows significantly thicker choriocapillaris measurements in this disease [1].

There are few studies describing OCTA features in CSCR patients [1-14], and to our knowledge, there is a paucity of such literature in the Indian population. The OCTA findings include dilated capillaries [1], abnormal vessels, dark areas, dark spots [3], abnormal blood flow [4], avascular areas corresponding to a detached retina [4], and a distinct choroidal neovascular membrane (CNVM) [2]. This study aimed to determine OCTA parameters in Indian patients with CSCR.

## Materials and methods

A cross-sectional study on Indian patients having unilateral or bilateral involvement with CSCR was conducted at the Department of Ophthalmology, Guru Nanak Eye Centre, Maulana Azad Medical College, New Delhi. The period of this study extended from January 2021 to December 2021. The study was conducted in compliance with the tenets of the Declaration of Helsinki and was approved by the Institutional Ethical Committee vide letter number F. 1/IEC/MAMC/(82/10/2020/No. 164 dated 14/01/2021. Patients with the best-corrected visual acuity (BCVA) of less than 6/60 were excluded to ensure reliable results for color vision (CV) and contrast sensitivity (CS). Additionally, patients with other significant diseases such as glaucoma, uveitis, media opacity, optic atrophy, retinal disease, refractive error of more than three dioptres, allergy to fluorescein sodium, or renal dysfunction were also excluded.

A history of ocular symptoms such as a diminution of vision, metamorphopsia, decreased CS, and defective CV and their duration were obtained. A personal history to identify risk factors such as smoking, sleep apnea, hypertension, and steroid intake; any systemic, renal, or liver disease; and allergy to fluorescein dye was elicited.

All patients provided written informed consent. They were examined for visual acuity (VA) on Early Treatment Diabetic Retinopathy Study (ETDRS) Charts at 4 m under uniform illumination. We performed a retinoscopy to prescribe appropriate refractive correction and recorded the BCVA. The intraocular pressure (IOP) was measured with an applanation tonometer. We studied CV on Ishihara color plates (38th edition, 2012) and the Farnsworth D-15 test. CS was assessed using Pelli Robson Charts (Haag-Streit Service, Inc., Ohio, USA). After dilating pupils, we examined the fundus and obtained color photographs on a fundus camera (VISUPAC and FF450plus, Carl Zeiss Meditec Inc., Dublin, California, USA). We performed FFA to look for dye leaks and pooling and ICGA to look for hypercyanescence.

We used spectral domain-OCT (RS-3000, Software NAVIS-EX) to examine central foveal thickness (CFT) and subfoveal choroidal thickness (SFCT) using the ETDRS grid including nine quadrants within 6 mm of the foveal center. The CFT was measured as the distance between the hyperreflective line corresponding to RPE and the internal limiting membrane (ILM). We measured the SFCT as the vertical distance from the RPE’s outer border to the sclera’s inner border [5]. The height of neurosensory detachment (NSD) and pigment epithelial detachment (PED) was measured manually using the supplied software. Subretinal fluid (SRF) height was measured from the vertical distance from the RPE line to the external limiting membrane (ELM) [5]. We scrutinized the OCT scans for intactness of the inner segment/outer segment (IS/OS) junction, RPE irregularities, and a double layer sign, constituted by a hyporeflective middle layer between the RPE and an intact or slightly thickened Bruch’s membrane. The eyes of symptomatic patients showing the presence of NSD and/or PED were denominated to have CSCR [6].

The OCTA (spectral-domain OCT; RS-3000 LITE advanced NIDEK) 3 x 3 mm scan, centered at the fovea, was used to study the FAZ, retino-choroidal capillaries, and VD, as well as determine the presence and location of the CNVM. Using the manufacturer’s software, we collected and analyzed the choriocapillaris image at the level of RPE/BM (i.e., at depth starting at 4 µ below RPE/BM till 32 µ below RPE/BM). We captured choroid image at the level of RPE/BM (i.e., at depth starting at 25 µ below RPE/BM till 63 µ below RPE/BM) [7]. The choriocapillaris abnormalities were classified into different patterns according to morphology. Some eyes showed distinctive hyperintense signals on OCTA signifying a hyperperfusion pattern. Other eyes recorded hyposignals and standard signals. We measured VD in the superficial capillary plexus (SCP), deep capillary plexus (DCP), outer retina (OR), outer retinal choriocapillaris (ORCC), choriocapillaris, and choroid. Images with a signal strength index of more than 90% were considered.

The primary outcome parameter was OCTA features in CSCR patients. The secondary outcome parameters were posterior-segment OCT features and the BCVA.

We compared the ocular, OCT, and OCTA values of affected eyes with those of the fellow eyes of our study participants.

Sample size calculation

According to the available literature (Chan et al.) [1], taking the percentage of patients showing dilated capillaries on OCTA in CSCR patients as 80.7%, at a 95% confidence level, and with a relative error of 20%, the sample size was estimated at 24. However, we included 52 eyes of 40 CSCR patients.

Statistical analysis

The collected data were entered in Microsoft Excel (Microsoft Corp., Redmond, WA, USA) and statistical analysis was performed using SPSS version 25.0 Software (IBM Corp., Armonk, NY, USA) in both primary and secondary outcomes. Each variable’s normality was assessed using the Kolmogorov-Smirnov and Shapiro-Wilk tests. Quantitative data were expressed as mean with standard deviation or median with interquartile range. The differences were determined using Student’s t-test and Mann-Whitney U test. Qualitative data was expressed in percentages, and the difference between the proportions was tested using the chi-square and Fisher’s exact test. P-values less than 0.05 were considered statistically significant.

## Results

We examined 52 eyes of 40 patients with acute or chronic CSCR. These included 32 (80%) males and eight (20%) females with a mean age of 39.3 ± 6.1 (24-49) years. The right eye was involved in 11/40 (27.5%) patients, the left eye was involved in 17/40 (42.5%) patients, and both eyes were involved in 12/40 (30%) patients. The mean duration of ocular symptoms was 374.7 ± 657.3 (7-2,920) days.

Out of 40 patients, 27/40 (67.5%) did not have any systemic disease, 7/40 (17.5%) had hypertension, 2/40 (5%) had diabetes, 1/40 (2.5%) had an allergy, 1/40 (2.5%) had joint pains, 1/40 (2.5%) had psoriasis, and 1/40 (2.5%) was menopausal. Among our study participants, 4/40 (10%) were teachers by occupation, 4/40 (10%) were tailors, 4/40 (10%) were shopkeepers, 4/40 (10%) were homemakers, 4/40 (10%) were carpenters, 2/40 (5%) were electricians, 2/40 (5%) were printers, 2/40 (5%) were businessmen, 2/40 (5%) were managers, 1/40 (2.5%) was a goldsmith, 1/40 (2.5%) was a salesman, 1/40 (2.5%) was a professor, 1/40 (2.5%) was a driver, 1/40 (2.5%) was a business administration student, 1/40 (2.5%) was an architect, 1/40 (2.5%) was a civil engineer, 1/40 (2.5%) was a computer typist, 1/40(2.5%) was a bag exporter, 1/40 (2.5%) was a data entry operator, 1/40 (2.5%) was a lab worker, and 1/40 (2.5%) worked in a dispensary. The average working time for these patients was 8.7 ± 2.3 (5-16) hours. A total of 7/40 (17.5%) patients complained of sleep apnea, and 13/40 (32.5%) had a history of drug use, including systemic steroids for systemic allergy, psoriasis, and joint pains and local steroids for local allergy; ayurvedic drugs for allergy; anti-diabetic and anti-hypertensive medicines; oral contraceptive pills; local skin ointment; and poly serum for hair growth.

The patients had complaints of diminution of vision (29/40 (72.5%)), metamorphopsia (10/40 (25%)), scotoma (8/40 (20%)), photopsia (1/40 (2.5%)) and defective CV (1/40 (2.5%)) in the affected eyes. The mismatched number is because one subject had more than one complaint. The eyes not having any symptoms were labeled as “fellow eyes.” The average systolic blood pressure was 129.7 ± 16.3 (110-162) mmHg, the average diastolic blood pressure was 84.8 ± 10.5 (55-105) mmHg, and the average mean blood pressure was 99.7 ± 10.7 (76.7-124.0) mmHg. The mean value of random blood sugar was 84.1 ± 10.5 (55-105) mg/dL. Among 80 eyes (40 patients), 52/80 (65%) eyes were affected, and 28/80 (35%) eyes were unaffected by CSCR. Table 1 shows the quantitative values for ocular features of affected and fellow eyes.

**Table 1:** Quantitative values for ocular features of affected and fellow eyes. *: Mean values with range and median values with interquartile range; **: parametric data; ***: statistically significant difference BCVA: best-corrected visual acuity

Ocular features (mean or median values)^*^	Affected eyes	Fellow eyes	P-value; test applied
Distance BCVA (LogMAR)^**^	0.58 ± 0.32 (0-1.5)	0.19 ± 0.22 (0-0.8)	<0.001^***^; Unpaired t-test
Spherical equivalent of distance refractive error (dioptres)^**^	0.77 ± 1.39 (-5.5-3.25)	0.27 ± 1.40 (-4.75-2.0)	0.130; Unpaired t-test
Intraocular pressure (mmHg)	17.5 ± 3.0 (12.0-29.0)	17.3 ± 3.1 (12.2-29.0)	-
	17.3 (14.6-18.6)	17.2 (16.5-18.2)	0.453; Mann-Whitney U test
Contrast sensitivity	1.39±1.49 (0.05-2.0)	1.61 ± 0.23 (1.1-1.85)	-
	1.55 (1.18-1.7)	1.70 (1.50-1.83)	0.063; Mann-Whitney U test
Ishihara plates read	31.7 ± 14.0 (0-38)	33.0 ± 12.4 (0-38)	-
	38.0 (38.0-38.0)	38.0 (38.0-38.0)	0.812; Mann-Whitney U test

The mean refractive error in affected eyes was 0.77 ± 1.39 (-5.5 to 3.25) dioptres; 6/52 (11.5%) eyes had myopia. Further investigation revealed that these patients had bilateral myopia and used myopic glasses since childhood. Except for nine eyes, every eye could read all the Ishihara plates. Of these nine eyes, one could read 13 plates, two could read one plate, and six could not read even a single plate. All these eyes had a central scotoma. None of the patients was found to have a red-green CV defect on this test. Among affected eyes, the Farnsworth D-15 test diagnosed 40/52 (76.9%) eyes as having no CV defect, 2/52 (3.8%) eyes having moderate tritanopia, 1/52 (1.9%) eyes having severe tritanopia, 2/52 (3.8%) eyes having protanopia, and 7/52 (13.5%) eyes having an irregular CV defect. Out of the fellow 28 eyes, only one (1.9%) eye had mild tritanopia, and the involved eye of the same patient also had mild tritanopia.

Among the affected eyes, the Amsler grid showed a normal plot in 13/52 (25%) eyes, metamorphopsia in 25/52 (48%) eyes, and scotoma in 14/52 (27%) eyes. In fellow eyes, this test showed metamorphopsia in 1/28 (3.6%) eyes and a normal plot in 27/28 (96.4%) eyes. The fellow eyes having mild tritanopia and mild metamorphopsia were not the same.

The OCT scans showed higher values for CFT and SFCT in the affected eyes. Additionally, NSD and PED were seen. In the 3 x 3 mm macular scans, NSD was seen in 40/52 (77%) affected eyes; out of these 40 eyes, 39 had one NSD, one had two NSD, and 12/52 (23%) eyes did not have NSD. On OCT, the mean height of NSD/sub-retinal fluid in the affected eye was 163 ± 199 (0-1,155) µ (median and interquartile range was 118.0 (27.0-223.5) µ), and the mean length of NSD/sub-retinal fluid was 1,776 ± 1,889 (0-7,820) µ (median and interquartile range was 1,266.5 (223.0-2,803.5) µ). In 5/52 (9.6%) subjects, the height of NSD was more than CFT, and in these subjects, NSD was located in the parafoveal or perifoveal region.

In the 3 x 3 mm macular scans, PED was seen in 32/52 (61.5%) affected eyes; 25 had one PED, four had two PED, two had three PED, and one had four PED. On OCT, the mean height and mean length of PED/sub-RPE fluid in the affected eye were 102 ± 115 (0-482) µ (median and interquartile range was 80.0 (0.0-131.0) µ) and 435 ± 512 (0-2120) µ (median and interquartile range was 336.50 (0.0-650.0) µ).

There were RPE irregularities in 10/52 (19.2%) eyes, disruption of the IS/OS junction in 12/52 (23.0%), CNVM in 2/52 (3.8%), exudate in 2/52 (3.8%), and a double layer sign in 5/52 (9.6%) eyes. The fellow eyes showed no OCT change on a 3 x 3 mm macular OCT scan. Table 2 shows the mean values of OCT and OCTA parameters in affected versus fellow eyes.

**Table 2:** Mean values of optical coherence tomography and optical coherence tomography angiography parameters in affected eyes versus fellow eyes. *: Parametric data; **: statistically significant difference SFCT: sub-foveal choroidal thickness; CFT: central foveal thickness; FAZ: foveal avascular zone; SCP: superficial capillary plexus; DCP: deep capillary plexus; OR: outer retina; ORCC: outer retinal choriocapillaris; CC: choriocapillaris

OCT and OCTA parameter mean values	Affected eyes	Fellow eyes	Mean difference	P-value; t-test
CFT (µ)	300.3 ± 158.4	223.1 ± 28.4	77.2 ± 130	0.013^**^
SFCT (µ)^*^	258.5 ± 60.4	248.3 ± 64.0	10.2 ± 3.9	0.447
FAZ area (mm^2^)	0.40 ± 0.71	0.34 ± 0.23	0.06 ± 0.48	0.666
FAZ perimeter (mm)	41.8 ± 280.8	76.8 ± 391.2	-35 ± 110.4	0.646
FAZ circularity^*^	0.48 ± 0.12	0.47 ± 0.11	0.01 ± 0.01	0.716
VD SCP foveal	10.1 ± 11.9	9.2 ± 10.9	0.9 ± 1	0.741
VD SCP parafoveal	24.7 ± 14.6	25.3 ± 13.6	-0.6 ± 1	0.858
VD SCP perifoveal	32.6 ± 18.4	35.8 ± 18.1	-3.2 ± 0.3	0.458
VD SCP whole	25.4 ± 14.1	25.9 ± 13.6	-0.5 ± 0.5	0.879
VD DCP foveal	4.4 ± 7.8	3.8 ± 5.9	0.6 ± 1.9	0.723
VD DCP parafoveal	11.8 ± 10.4	14.4 ± 11.0	-2.6 ± 0.6	0.2991
VD DCP perifoveal	22.5 ± 15.9	25.5 ± 18.6	-3 ± 2.7	0.451
VD DCP whole	15.0 ± 11.5	16.5 ± 11.7	-1.5 ± 0.2	0.582
VD OR foveal	6.3 ± 9.3	14.0 ± 16.0	7.7 ± 6.7	0.008^**^
VD OR parafoveal	6.3 ± 8.8	17.8 ± 17.2	-11.5 ± 8.4	<0.001^**^
VD OR perifoveal	5.4 ± 5.0	12.1 ± 13.8	-6.7 ± 8.8	0.002^**^
VD OR whole	5.9 ± 6.8	14.3 ± 14.9	-8.4 ± 8.1	<0.001^**^
VD ORCC foveal	33.6 ± 19.7	37.2 ± 19.7	-3.6 ± 0	0.438
VD ORCC parafoveal	33.1 ± 17.5	37.0 ± 16.0	-3.9 ± 1.5	0.331
VD ORCC perifoveal	34.8 ± 15.3	38.3 ± 15.9	-3.5 ± 0.6	0.339
VD ORCC whole	33.7 ± 16.9	38.0 ± 16.5	-4.3 ± 0.4	0.277
VD CC foveal	30.7 ± 20.2	38.1 ± 20.2	-7.4 ± 0	0.122
VD CC parafoveal	29.7 ± 18.2	36.2 ± 18.0	-6.5 ± 0.2	0.130
VD CC perifoveal	30.0 ± 16.0	35.8 ± 17.3	-5.8 ± 1.3	0.137
VD CC whole	29.7 ± 17.5	36.3 ± 17.7	-6.6 ± 0.2	0.113
VD choroid foveal	29.4 ± 20.0	39.8 ± 21.0	-10.4 ± 1	0.032^**^
VD choroid parafoveal	29.6 ± 18.1	35.6 ± 19.5	-6 ± 1.4	0.173
VD choroid perifoveal	29.8 ± 16.6	34.9 ± 19.4	-5.1 ± 3.1	0.221
VD choroid whole	29.9 ± 17.5	35.5 ± 19.2	-5.6 ± 1.7	0.804

Among OCT and OCTA parameters, only the data for SFCT and FAZ circularity was found to be parametric. Hence, median values were calculated for all parameters, and the significance of the difference between the values of affected and fellow eyes was found. Table 3 shows the median values of OCT and OCTA parameters in affected versus fellow eyes.

**Table 3:** Median values of optical coherence tomography and optical coherence tomography angiography parameters in affected eyes versus fellow eyes *: Parametric data; **: statistically significant difference SFCT: sub-foveal choroidal thickness; CFT: central foveal thickness; FAZ: foveal avascular zone; SCP: superficial capillary plexus; DCP: deep capillary plexus; OR: outer retina; ORCC: outer retinal choriocapillaris; CC: choriocapillaris

OCT and OCTA parameter median values	Affected eyes	Fellow eyes	P-value; Mann-Whitney test
CFT (µ)	242.0 (196.0-349.0)	219.5 (204.0-238.0)	0.084
SFCT (µ)^*^	253.0 (219.5-291.5)	242.0 (200.0-269.5)	0.271
FAZ area (mm^2^)	0.32 (0.21-0.42)	0.31 (0.24-0.44)	0.916
FAZ perimeter (mm)	2.81 (2.20-3.43)	3.15 (2.37-3.44)	0.408
FAZ circularity^*^	0.51 (0.41-0.56)	0.47 (0.40-0.56)	0.720
VD SCP foveal	6.0 (2.0-14.0)	5.0 (2.5-9.5)	0.777
VD SCP parafoveal	19.5 (13.5-38.0)	29.5 (12.5-38.0)	0.972
VD SCP perifoveal	22.0 (19.0-53.0)	42.5 (18.0-53.5)	0.554
VD SCP whole	23.0 (14.0-38.0)	29.5 (14.0-34.0)	0.828
VD DCP foveal	1.0 (00-6.0)	1.0 (0.0-6.5)	0.936
VD DCP parafoveal	9.0 (4.0-18.5)	11.5 (5.5-22.0)	0.335
VD DCP perifoveal	17.5 (11.0-39.5)	20.5 (11.0-44.5)	0.552
VD DCP whole	12.0 (6.0-23.0)	14.0 (6.0-26.0)	0.545
VD OR foveal	2.5 (0.0-9.0)	8.0 (2.0-19.0)	0.009^**^
VD OR parafoveal	3.5 (1.0-9.5)	11.5 (3.0-25.5)	0.001^**^
VD OR perifoveal	5.0 (1.0-8.0)	7.0 (2.0-17.5)	0.058
VD OR whole	4.0 (1.0-8.0)	8.5 (3.5-17.5)	0.006^**^
VD ORCC foveal	25.5 (16.5-52.5)	38.0 (19.0-56.5)	0.377
VD ORCC parafoveal	24.0 (17.5-50.0)	42.5 (21.5-51.0)	0.296
VD ORCC perifoveal	35.0 (21.0-52.0)	46.0 (21.5-53.0)	0.180
VD ORCC whole	26.0 (18.0-51.0)	41.5 (21.0-53.5)	0.234
VD CC foveal	20.0 (14.0-51.5)	46.5 (18.5-56.5)	0.061
VD CC parafoveal	18.0 (15.5-50.0)	45.0 (17.5-53.0)	0.135
VD CC perifoveal	20.0 (17.0-48.0)	43.5 (18.5-53.0)	0.071
VD CC whole	19.0 (15.5-50.0)	47.0 (17.0-52.5)	0.096
VD choroid foveal	18.5 (14.0-50.0)	45.5 (16.0-58.5)	0.022^**^
VD choroid parafoveal	19.5 (14.0-49.5)	46.09 (13.5-53.5)	0.182
VD choroid perifoveal	24.5 (14.0-47.5)	45.5 (14.5-53.0)	0.095
VD choroid whole	20.5 (14.0-49.5)	46.5 (14.0-52.5)	0.184

The CSCR eyes had a thicker fovea and SFC. The FAZ area of affected eyes was larger, while the perimeter was smaller than that in the fellow eye. The values of circularity were not much different in affected and fellow eyes. In the affected eye, the VD in all the retino-choroidal layers including SCP, DCP, OR, ORCC, choriocapillaris, and choroid was lesser though it was significantly reduced in OR whole (p = 0.006) and foveal choroid (p = 0.022). The exception was VD in SCP foveal and DCP foveal, where the affected eye had a higher VD.

The FFA documented NSD in 33/52 (63.5%) affected eyes, PED in 16/52 (30.8%), RPE changes in 8/52 (15.4%), CNVM in 3/52 (5.8%), and exudate in 1/52 (1.9%).

We performed ICGA in all our patients and found increased vascularity and vessel dilatation depicted by choroidal hypercyanescence at sites corresponding to the NSD and PED and in the entire choroid of the affected eye.

From counting the number of eyes showing NSD and PED on different imaging techniques, it is visible that the central OCT scans showed these changes more frequently than FFA.

While the fellow eyes had a normal 3 x 3 mm macular OCT scan, the FFA in these eyes showed RPE changes in 8/28 (15.3%), PED in 3/28 (5.8%), NSD in 3/28 (5.8%), and scar in 1/28 (1.9%); 15/28 (28.8%) fellow eyes had a normal FFA. Notably, on ICGA, the fellow eyes showed choroidal hypercyanescence. Table 4 shows the distribution of eyes for OCTA features in affected and fellow eyes.

**Table 4:** Distribution of eyes for optical coherence tomography angiography features in affected and healthy eyes. *Fisher’s exact test. OCTA: optical coherence tomography angiography; FAZ: foveal avascular zone; NSD: neurosensory detachment; PED: pigment epithelial detachment; CNVM: choroidal neovascular membrane; CCI: choriocapillaris island

OCTA features	Number of affected eyes	Number of fellow eyes	Chi-square test/Fisher’s exact test^*^
Enlarged/distorted FAZ	19/52 (36.53%)	5/28 (17.85%)	0.082
Dark areas (NSD/PED)	44/52 (84.61%)	7/28 (25.0%)	<0.001
Dark spots (PED)	3/52 (5.76%)	0 (0%)	0.548^*^
Abnormal vessels (dilated vessels/CNVM)	50 (96.15%)	22/28 (78.57%)	0.125
CCI	9/52 (17.30%)	3/28 (10.71%)	0.526^*^
Normal scan	0 (0%)	4/28 (14.28%)	0.013^*^

The dark areas generally correspond to the NSD and the dark spots to the PED [3]. The choriocapillaris island (CCI) is an island with a detectable choriocapillaris flow surrounded by an area of undetectable or diminished flow underneath the area of NSD [8]. The OCTA showed positive findings related to the CSCR in all 28/28 (100%) fellow eyes. The OCTA showed a higher number of CSCR features in affected eyes than fellow eyes; however, this number was significantly higher for NSD (p < 0.001). Table 5 shows OCTA features in the eyes affected with CSCR and fellow eyes.

**Table 5:** Optical coherence tomography angiography findings in affected and healthy eyes. OCTA: optical coherence tomography angiography; RE: right eye; LE: left eye; BE: both eyes; SCP: superficial capillary plexus; DCP: deep capillary plexus; OR: outer retina; ORCC: outer retinal choriocapillaries; CC: choriocapillaries Enlarged/distorted foveal avascular zone = 1; dark area (neurosensory detachment/pigment epithelial detachment) = 2; dark spots (pigment epithelial detachment) = 3; abnormal vessels (dilated vessels/choroidal neovascular membrane) = 4; choriocapillaries island = 5; normal = 6

Serial number of patients	Eye affected	OCTA features of affected eyes	OCTA features of fellow eyes
1	LE	1, 2 (DCP, OR, ORCC)	6
2	RE	1, 2 (OR, ORCC), 4	1,4
3	BE	1,2 (OR, ORCC, CC, Ch), 3 (ORCC, CC, Ch), 4 (ORCC, CC, Ch), 5 (ORCC, CC, Ch)	
	BE	2 (ORCC, CC, Ch), 3 (ORCC, CC, Ch), 4 (ORCC, CC, Ch), 5 (ORCC, CC, Ch)	
4	RE	1, 2 (OR, ORCC, CC, Ch), 4 (CC, Ch)	6
5	RE	1, 2 (DCP, OR), 3 (ORCC, CC, Ch), 4 (CC, Ch)	4 (CC, Ch)
6	BE	1, 2 (DCP, OR), 4 (ORCC, CC, Ch)	
	BE	1, 2 (DCP, OR, ORCC), 4 (CC, Ch)	
7	RE	1, 2 (DCP, OR, ORCC), 4 (CC, Ch)	1, 4 (CC, Ch)
8	RE	1, 2 (DCP, OR, ORCC), 4 (ORCC, CC, Ch), 5 (CC, Ch)	4 (CC, Ch)
9	LE	4 (CC, Ch)	4 (CC, Ch), 5 (CC, Ch)
10	BE	2 (OR, ORCC), 4 (CC, Ch), 5 (CC, Ch)	
	BE	4 (CC, Ch)	
11	LE	2 (DCP, OR), 4 (CC, Ch)	4 (CC, Ch)
12	BE	2 (DCP, OR, ORCC, CC, Ch), 4 (CC, Ch)	
12	BE	2 (DCP, OR, ORCC, CC, Ch), 4 (CC, Ch)	
13	RE	4 (CC, Ch)	4 (CC, Ch)
14	LE	1, 2 (DCP, OR, ORCC), 4 (CC, Ch)	6
15	RE	2 (DCP, OR), 4 (ORCC, CC, Ch),	6
16	RE	1, 2 (DCP, OR, ORCC), 4 (CC, Ch)	2 (OR, ORCC, CC, Ch), 4 (ORCC, CC, Ch)
17	LE	2 (DCP, OR), 4 (CC, Ch)	1, 2 (DCP, OR), 4 (CC, Ch)
18	LE	2 (DCP, OR, ORCC, CC, Ch), 4 (CC, Ch)	4 (ORCC, CC, Ch)
19	RE	1, 4 (CC, Ch)	4 (CC, Ch), 5 (CC)
20	LE	4 (CC, Ch)	4 (CC, Ch)
21	BE	2 (OR, ORCC), 4 (CC, Ch), 5 (ORCC)	
	BE	2 (DCP, OR, ORCC, CC, Ch), 4 (CC, Ch)	
22	BE	2 (DCP, OR, ORCC, CC, Ch), 4 (CC, Ch)	
	BE	2 (OR), 4 (ORCC, CC, Ch)	
23	LE	4 (CC, Ch)	4 (CC, Ch)
24	LE	1, 2 (DCP, CC, Ch), 4 (CC, Ch)	4 (CC, Ch)
25	BE	2 (DCP, OR), 4 (ORCC, CC, Ch),	
	BE	1, 2 (OR), 4 (ORCC, CC, Ch)	
26	LE	1, 2 (DCP, OR, ORCC), 4 (ORCC, CC, Ch), 5 (CC, Ch)	2 (OR), 4 (ORCC, CC, Ch)
27	LE	4 (CC, Ch)	4 (CC, Ch)
28	RE	2 (DCP, OR, ORCC), 4 (ORCC, CC, Ch), 5 (ORCC, CC, Ch)	4 (CC, Ch)
29	LE	2 (DCP, OR, ORCC, CC, Ch), 4 (DCP, OR, ORCC, CC, Ch), 5 (CC, Ch)	2 (DCP, OR), 4 (ORCC, CC, Ch)
30	BE	2 (DCP, OR, ORCC), 4 (CC, Ch)	
	BE	2 (DCP, OR, ORCC), 4 (CC, Ch)	
31	LE	2 (DCP, OR, ORCC), 4 (ORCC, CC, Ch)	1, 2 (DCP, OR, ORCC), 4 (CC, Ch)
32	RE	1, 2 (SCP, DCP)	1
33	LE	2 (OR, ORCC), 4 (CC, Ch)	4 (CC, Ch)
34	LE	1, 2 (DCP, OR, ORCC), 4 (CC, Ch)	2 (ORCC), 4 (CC, Ch), 5 (CC, Ch)
35	BE	1, 2 (DCP, OR, ORCC), 4 (CC, Ch)	
	BE	1, 2 (DCP, OR, ORCC), 4 (CC, Ch)	
36	BE	2 (DCP, OR, ORCC), 4 (ORCC, CC, Ch)	
	BE	2 (DCP, OR, ORCC), 4 (ORCC, CC, Ch), 5 (ORCC, CC, Ch)	
37	LE	2 (DCP, OR), 4 (ORCC, CC, Ch)	4 (CC, Ch)
38	BE	2 (DCP, OR), 4 (CC, Ch)	
	BE	2 (DCP, OR), 4 (ORCC, CC, Ch)	
39	LE	2 (DCP, OR), 4 (ORCC, CC, Ch)	2 (CC, Ch)
40	BE	2 (DCP, OR), 4 (ORCC, CC, Ch)	
	BE	4 (ORCC, CC, Ch)	

Table 5 shows different OCTA findings, including enlarged/distorted FAZ, dark area (NSD/ PED), dark spot (PED), abnormal vessels (dilated vessels/CNVM) and CCI, and their location in different retino-choroidal layers. Table 4 and Table 5 show that OCTA detected features of CSCR even in fellow eyes.

## Discussion

There are a few studies on OCTA findings in CSCR patients transcended by the fact that there is a paucity of this literature in Indian patients [1-14]. Liu et al. examined 152 eyes of 144 subjects, and Seo et al. examined 68 eyes of 68 subjects, while the number of eyes examined in other studies was lesser than ours [1-14]. The mean age of patients in our study was 39.3 ± 6.1 years which was lesser than those studied by others [1-14]. Co-existing systemic diseases in CSCR subjects have been mentioned infrequently [3,4,7,8,10,11]. Six of the 10 subjects in the study by Maftouhi et al. had a history of steroid use [2]. In the study by Mandadi et al., 12 subjects had hypertension, four had type 2 diabetes, two had a smoking history, and one had bronchial asthma [9]. Other studies have not commented on the nature of co-existing systemic disease. In our study, 27/40 patients had no systemic disease while others had conditions such as hypertension, diabetes, allergy, joint pains, psoriasis, and menopause. Table 6 shows the demographic, clinical, and ocular features of subjects examined in different studies.

**Table 6:** Demographic, clinical, and ocular features of central serous chorioretinopathy subjects examined in different studies. BCVA: best-corrected visual acuity; OCT: optical coherence tomography; OCTA: optical coherence tomography angiography; CSCR: central serous chorioretinopathy; SFCT: sub-foveal choroidal thickness; NSD: neurosensory detachment; PED: pigment epithelial detachment; OS: outer segment; FIPED: flat irregular pigment epithelial detachment; CNVM: choroidal neovascular membrane; CCI: choriocapillaris island; RPE: retinal pigment epithelium; FAZ: foveal avascular zone

Authors	Number of subjects	Mean age (years)	BCVA (LogMAR)	OCT findings	OCTA findings
Chan et al. [1]	26 eyes of 21 subjects	47.0 ± 7.9	+1.0 to -0.1	SFCT of 428 ± 70 µ	High signal intensity in 26 eyes and dilated CC (25.8 ± 1.2 µ) in 21 eyes
Maftouhi et al. [2]	12 eyes of 10 subjects (chronic CSCR)	54.6	+0.2	SFCT of 400 microns, NSD in 9 eyes, PED in 2 eyes, and elongation of OS in 5 eyes	CNVM in OR slab of 7 eyes; normal retinal and choroidal circulation
Costanzo et al. [3]	33 eyes of 32 subjects; 9 (acute CSCR) and 24 (chronic disease)	54.8 ± 10.4	0.23 ± 0.25	NSD in 17 eyes, and PED in 5 eyes	Abnormal flow in OR in 6 eyes, dark areas in 19 eyes, dark spots in 7 eyes, and abnormal choroidal vessels in CC layer in 12 eyes
Feucht et al. [4]	11 eyes of 10 subjects	Did not mention	Did not mention	NSD in 11 eyes	NSD in 4 eyes, and irregular flow pattern in CC in 5 eyes
Seo et al. [5]	68 eyes of 68 subjects	48.2 ± 9.5	Did not mention	NSD in 68 eyes	Three flow patterns in CC were seen: hyper-perfusion (in resolved CSCR), mixed perfusion (in active CSCR), and normal perfusion
Bansal et al. [7]	43 eyes of 26 subjects with chronic disease	45.6 ± 8.5	Did not mention	NSD in 26 eyes, intra-retinal fluid in 6 eyes, PED in 14 eyes, and FIPED in 10 eyes	CNVM in 9 eyes
Estawro et al. [8]	25 eyes of 25 subjects	40.6 ± 9.7	+0.1 to +0.7	CFT of 417.7 ± 106.1 µ; SFCT of 433.3 ± 94.6 µ, NSD in 25 eyes	CCI in all 25 eyes
Mandadai et al. [9]	40 eyes of 40 subjects	54.9 ± 9.9	0.36 ± 0.28	CFT of 267.4 ± 6,109.5 µ, SFCT of 412 ± 103 µ, NSD in 30 eyes, PED in 42 eyes, hyperreflective dots in choroid in 30 eyes	CC voids in 40 eyes, CNVM in 40 eyes
Liu et al. [10]	152 eyes of 144 subjects	51 ± 8.86	0.69 ± 0.13 in the non-CNVM group; 0.39 ± 0.23 in the CNVM group	SFCT of 260 ± 57 µ in the non-CNVM group and 239 ± 25 µ in the CNVM group, FIPED in 35 eyes	CNVM in 11 eyes
Cakir et al. [13]	101 eys of 78 subjects	51	0.71	NSD, RPE atrophy	Decreased OCTA signal in the SRF area; no CC OCTA changes in altered RPE adjacent to atrophy
Our study	52 eyes of 40 subjects	39.3 ± 6.1	0.58 ± 0.32	CFT of 300 ± 158 µ; SFCT of 259 ± 60 µ; NSD in 40 eyes, PED in 40 eyes,	Distorted FAZ in 19 eyes, dark area in 44 eyes, dark spots in 3 eyes, abnormal vessels in 30 eyes, and CCI in 9 eyes

Only the subjects in the studies by Chan et al. and Liu et al. had a worse BCVA than our subjects [1,10]. The mean refractive error was 20.75 ± 0.31 dioptres in the study by Chan et al. [1], while it was 0.77 ± 1.39 (-5.5 to 3.25) dioptres in our study. Other authors did not comment on the refractive errors of their subjects. Despite CSCR being a central involvement, the Amsler test has not been done frequently. Baran et al. found a significantly higher incidence of metamorphopsia in 67.7% of CSCR eyes than the fellow eyes (p = 0.0) [15]. We found metamorphopsia in 25/52 (48%) eyes and scotoma in 14/52 (27%) eyes; 13/52 (25%) eyes had a normal plot. Of the fellow 28 eyes, only one (1.9%) eye had mild metamorphopsia.

Very few authors compared the IOP, CS, and CV of the affected eye with the fellow eye. Using the Farnsworth-Munsell L’Anthony-40 hue (FM 40) test, Baran et al. found a color discrimination defect in 48.4% of CSCR eyes and 54.8% of fellow eyes. On Cambridge charts, the mean CS score of the eyes with CSCR was significantly lower than fellow eyes (146.7 ± 86.3 vs. 200.7 ± 104.3; p = 0.004) [15]. Saad et al. performed the Farnsworth D-15 dichotomous test and observed CV defects in 73.3% of subjects; 50% of these had a tritan defect [16]. We found increased IOP, reduced CS, and defective CV in the affected eye. There was a tritan defect in 3/52 (5.8%) eyes, a protan defect in 2/52 (3.8%) eyes, and an irregular CVD in 7/52 (13.5%) eyes; 1/52 (1.9%) fellow eyes had mild tritanopia. Unlike other authors, we conducted additional testing of CV with the Ishihara test and found that out of 38 plates, the mean number of plates read by affected eyes was 31.7 ± 14.0 and by fellow eyes was 33.0 ± 12.4. None of our patients had a red-green CV defect. We found the mean CS value in affected eyes was 1.39 ± 1.49 while it was 1.61 ± 0.23 in fellow eyes. These findings show that even fellow eyes have a defective CV and a diminished CS, as shown in Table 1. We also calculated the statistical significance of differences in the clinical features of affected and fellow eyes.

While we found more than one NSD and PED in CSCR eyes, these findings have been rarely commented upon [2]. In the central fundus, the NSD and PED were more frequently picked up on OCT scans than FFA. The 55-degree fundus imaging in FFA and ICGA showed findings suggestive of CSCR in the area peripheral or adjacent to the central 3 x 3 mm fundus; thus, 3 x 3 mm macular OCT scans may not be sufficient in ruling out the affection of the remaining fundus.

The authors observed OCTA findings such as dilated capillaries, distorted FAZ, abnormal flow patterns, dark areas, dark spots, abnormal choroidal vessels, CNVM, CCI or voids, and reduced OCTA signals in areas of SRF [1-14]. We observed similar OCTA changes in the involved eye. Very few authors have reported OCTA findings in fellow eyes of their study subjects. Chan et al. showed a high-intensity signal pattern in the OCTA in the contralateral clinically unaffected eye [1]. Among 40 fellow eyes of CSCR eyes having CNVM, Mandadi et al. found that 25 had a flat irregular PED and nine had CNVM. As OCTA has the distinct advantage of detecting CNV earlier than conventional imaging, these authors highlighted the importance of doing OCTA in fellow eyes to monitor CNVM activity [9]. Notably, we found that OCTA showed positive findings related to the CSCR in all 28/28 (100%) fellow eyes, while FFA showed involvement in only 13/28 (46.4%) fellow eyes.

In addition to studying qualitative features in OCTA, we found values for quantitative parameters including mean FAZ area 0.40 ± 0.71 mm^2^, perimeter 41.8 ± 280.8 mm, and circularity 0.48 ± 0.12. In the CSCR eye, the FAZ area was larger while the perimeter was smaller than that in the fellow eyes. The values of circularity were not much different in affected and fellow eyes. We can explain different affection of area and perimeter based on the formula used for their calculation. We calculate the area of a circle as 3.14 × r^2^ and the perimeter as 2 x 3.14 x 7 r. When r = 1, the values of the perimeter are more than that of the area; when r = 2, the values of the area and perimeter are equal. When r = 3 and onwards, the magnitude of the area becomes higher than that of the perimeter, so we infer that the affected eye having higher values for the area but lower for the perimeter compared to the fellow eye reflects a slightly advanced stage of the disease.

We found the mean VD in SCP whole was 25.4 ± 14.1, in DCP whole was 15.0 ± 11.5, in OR whole was 5.9 ± 6.8, in ORCC whole was 33.7 ± 16.9, in choriocapillaris whole was 29.9 ± 17.5, and in choroid whole was 29.9 ± 17.5. The VD of all retino-choroidal layers was lesser in affected than the fellow eye, most probably due to the pressure effect of the NSD or PED, which caused a compromised blood flow in these layers or due to abnormal choriocapillary flow dynamics [3,5]. The affected eye had however a higher VD in the central 1 mm of SCP and DCP (SCP foveal and DCP foveal) and appears to have escaped this pressure effect. The previous studies rarely provided quantitative values for OCTA parameters in CSCR.

Limitations

The limitation of this study was that the FF450plus fundus camera provided only a central 55-degree fundus view, and equipment used by us for OCTA obtained only 3 mm × 3 mm scans. The periphery of the fundus may have more CSCR lesions that could not be examined.

## Conclusions

The incidence of CSCR occurs at a lower age in Indian patients. The CSCR eyes had a thicker fovea and sub-foveal choroid than fellow eyes. The FAZ area of affected eyes was more than the fellow eye, while circularity was not much affected. The OCTA scans showed that the VD of all the retino-choroidal layers was reduced. We found OCT to be more sensitive than FFA in detecting the presence of disease in the affected eye. OCTA was also more sensitive than FFA in detecting the presence of disease in both the affected and fellow eye. Additionally, imaging at sites peripheral to 3 x 3 mm central scan may show a wider involvement implying that the disease of CSCR may not be restricted to the central fundus only. This highlights the role of multimodal imaging in CSCR. Seeing the involvement of fellow eyes on the CV, CS, and Amsler tests, and findings on FFA, ICGA, and OCTA, we may conclude that at initial stages, the fellow eyes may show a partial affection. However, CSCR may manifest bilaterally in later stages, thus follow-up in fellow eyes is extremely important.
